# Modeling the potential effect of cabamiquine-pyronaridine for chemoprevention against *Plasmodium falciparum* malaria

**DOI:** 10.1128/aac.00434-26

**Published:** 2026-10-08

**Authors:** Lucy C. Okell, Perrine Courlet, Filippo Venezia, Claude Oeuvray, Claudia Demarta, Thomas Spangenberg, Joseph Okebe

**Affiliations:** 1 MRC Centre for Global Infectious Disease Analysis, School of Public Health, Imperial College London 4615, London, United Kingdom; 2 Merck Institute for Pharmacometrics, Ares Trading S.A. (an affiliate of Merck KGaA, Darmstadt, Germany) 2792 , Lausanne, Switzerland; 3 The Healthcare Business of Merck KGaA 2792 , Darmstadt, Germany; 4 Global Health R&D of Merck Healthcare, Ares Trading S.A. (an affiliate of Merck KGaA, Darmstadt, Germany), Eysins, Switzerland; The Children's Hospital of Philadelphia, Philadelphia, Pennsylvania, USA

**Keywords:** cabamiquine, transmission modeling, pharmacokinetic-pharmacodynamic modeling, malaria

## Abstract

Preventive treatment (chemoprevention) is a key intervention for reducing the burden of *Plasmodium falciparum* malaria in children. The impact of the current widely used drug combination, sulfadoxine-pyrimethamine-amodiaquine (SP-AQ), is challenged by emerging drug resistance and adherence to multi-day dosing. Cabamiquine is a novel antimalarial with activity against liver- and blood-stage malaria parasites, administered as a single dose, and under development in combination with pyronaridine. We used mathematical modeling to translate early phase 1 clinical results into estimates of its potential impact as malaria chemoprevention. Pharmacokinetic-pharmacodynamic (PKPD) models of cabamiquine and pyronaridine were combined, assuming Bliss independence, to estimate time-varying protection against infection. PKPD results were incorporated into an established individual-based malaria transmission model to estimate the impact of seasonal malaria chemoprevention (SMC) across settings with differing transmission and seasonality. Cabamiquine-pyronaridine was predicted to provide protection of >95% for approximately 4 weeks, slightly outperforming SP-AQ even in children who fully adhere to SP-AQ treatment. Six weeks after treatment, cabamiquine-pyronaridine protection dropped for the regimens containing <200 mg cabamiquine, but the combination could still outperform SP-AQ at doses ≥200 mg. If deployed as SMC, a longer duration of protection may enable fewer, more widely spaced treatment cycles while maintaining impact, improving projected cost-effectiveness. Cabamiquine-pyronaridine shows strong potential as a next-generation single-dose chemoprevention regimen, supporting continued clinical development and evaluation.

## INTRODUCTION

Administering antimalarial drugs to children at risk of malaria to prevent infection and illness is proving a highly effective strategy to reduce *Plasmodium falciparum* malaria burden (1). In the Sahel region of Africa, millions of children receive seasonal malaria chemoprevention (SMC) each year with the drug combination sulfadoxine-pyrimethamine-amodiaquine (SP-AQ) (2). In trials, SP-AQ was shown to prevent around 75%–90% of clinical episodes when children are given monthly doses during the high-transmission season (3). Perennial malaria chemoprevention (PMC, formerly known as intermittent preventive treatment in infants), intermittent preventive treatment in school children, and post-discharge chemoprevention (for patients recovering from severe malarial anemia) are also recommended by the World Health Organization (WHO), including in areas with more perennial transmission (1). The largest amount of evidence on chemoprevention efficacy in children comes from trials of SP-AQ (used for SMC) and trials of SP (used for PMC). Alternative regimens such as artesunate-amodiaquine and dihydroartemisinin-piperaquine have also been trialed. In June 2022, the WHO chemoprevention guidelines were considerably broadened to give countries flexibility to choose between different drugs, target age groups, seasonal timings, and number of rounds. It is recommended to use a different drug for chemoprevention from the first-line treatment for symptomatic malaria. Mass drug administration (MDA), where a large part of the population is given preventive treatment, is also recommended in some settings (1).

While SP-AQ is a cost-effective and successful treatment in the Sahel, in Eastern, Southern, and parts of Central Africa, parasite populations have higher levels of drug resistance to SP, mediated by mutations in the *dhps* and *dhfr* genes (4–6). Here, the efficacy of chemoprevention with SP-AQ remains uncertain, with mixed evidence to date. However, many areas in these regions also experience extremely high malaria burden (7) and would benefit from chemoprevention. Furthermore, SP-AQ is given as three doses over 3 days, with only the first dose directly observed by a healthcare worker, meaning that imperfect adherence in routine settings may contribute to a reduced impact (8). In areas with perennial transmission, SP-AQ would need to be given for most of the year, and there are limited data on safety and toxicity for more than five to six doses per year (3). There is therefore a need for new drugs for chemoprevention in the future.

Cabamiquine (previously known as M5717, DDD107498) is a new drug candidate with a novel mode of action that rapidly blocks parasite protein synthesis by inhibiting the eukaryotic elongation factor 2, leading to activity against multiple parasite life stages, including gametocytes and liver-stage parasites (9), unlike other antimalarials which often target only the blood stages of the parasite lifecycle (10). It is also administered as a single oral dose and recently completed phase 2a trials (NCT05689047 and NCT05974267) where it has been combined with pyronaridine to prevent drug resistance (11, 12). As such, it offers promise to provide protective effects against new infection if used for chemoprevention in children or other vulnerable groups, or for MDA. Pharmacokinetic and pharmacodynamic modeling of cabamiquine and pyronaridine have been separately undertaken, using results from phase 1 dose escalation studies. However, these trials did not directly test chemoprevention, since the drug was given either shortly before or after malaria infection, whereas for preventive treatment we would like to know what protection the drug regimen offers over 4–6 weeks after dosing.

In this analysis, we use a mathematical modeling framework to estimate (i) the pharmacodynamics of the combination of cabamiquine and pyronaridine, (ii) the long-term duration of protection, that is, the probability that the drug regimen would protect a person from malaria infection up to 45 days after treatment, and (iii) the potential public health impact of deploying cabamiquine-pyronaridine for seasonal malaria chemoprevention. While there are limitations of basing such predictions on early phase 1 data, these results can give an early indication of potential prophylactic efficacy to help inform decisions on whether to trial the drug regimen for chemoprevention, rather than only for cure of symptomatic malaria. Our results are intended to be validated in future against phase 2 and phase 3 data to assess the utility of our methods for estimating prophylactic efficacy, which may be useful for future compounds in development.

## MATERIALS AND METHODS

### Estimating chemoprevention efficacy using pharmacokinetic-pharmacodynamic (PKPD) modeling

We combined previously developed PKPD models of cabamiquine (13) and pyronaridine (14) to estimate their potential efficacy at preventing new infections when used for chemoprevention. The data on which the models are based and their full methods are described in the previous publications. Here, we give a brief summary. The cabamiquine PKPD model was previously developed by analyzing two phase I dose-escalation placebo-controlled human malaria infection (CHMI) trials in healthy adult men. A population PK model successfully characterized the concentration-time profile of cabamiquine in 101 adults. Parasite density over time in the liver and blood was simulated using a pre-existing within-host model calibrated to pooled parasitaemia data from 15 trials (15). A cabamiquine pharmacodynamic model describing both liver- and blood-stage killing effects of the drug was then jointly fitted to the phase I trials, which used either an induced blood-stage malaria model to directly infect participants with blood-stage parasites 8 days before receiving cabamiquine (*n* = 22 participants), or a sporozoite challenge model to infect participants 2 hours or 96 hours before cabamiquine treatment (*n* = 39 participants). The pyronaridine PKPD model was similarly developed based on CHMI trials, where 10 volunteers received infected erythrocytes on day 0 and were administered different single oral doses of pyronaridine on day 8. PK and PD parameters were estimated, assuming the drug only affects the blood-stage parasites.

For the current work, we combined the PKPD models of cabamiquine and pyronaridine, enabling quantitative simulation of their joint capacity to prevent the establishment of new infections under chemoprevention. Consistent with available evidence, we assumed no synergistic or antagonistic interaction on blood-stage parasites, such that the combined effect was represented as an additive Bliss-independent interaction with a negligible-overlap approximation. Based on the reported potentiation of cabamiquine liver-stage activity in the presence of pyronaridine (16), combination treatment was represented by a threefold reduction in the apparent cabamiquine EC50. This parameter adjustment captured the enhanced liver-stage effect of the combination while retaining a mechanistically consistent model architecture. The schematic representation of the integrated PK/PD model is detailed in Fig. S4.

Using the cabamiquine-pyronaridine combined PKPD models, we estimated the probability that the drug combination would prevent new infections at any given time after treatment, conditional upon being exposed at that time. We simulated individuals who were malaria-free at the time of receiving the drug and were exposed to sporozoites at different times post-treatment (days 0–60), using 1,000 stochastic realizations per scenario to allow for interindividual variability in PK and PD parameters. We simulated the standard dose of pyronaridine, 720 mg, and a range of doses of cabamiquine: 60 mg, 100 mg, 200 mg, 330 mg, 660 mg. Parasite clearance was defined as reaching a total parasitemia in the body of <1 parasite per microliter. In the case where chemoprevention failed to prevent infection, we assumed that the infection would become detectable when parasite density crossed a threshold of 10 parasites per microliter, in line with the approximate microscopy detection threshold (17). We assumed one parasite exposure per person during follow-up at this initial stage in order to calculate chemoprevention efficacy conditional on a single exposure.

### Transmission modeling and the impact of cabamiquine-pyronaridine chemoprevention

An existing, open-source, individual-based model of malaria transmission, *malariasimulation,* developed at Imperial College London (ICL), UK, was used to model the potential impact of cabamiquine-pyronaridine chemoprevention in different scenarios. The model has been described in detail elsewhere (18–23). In brief, the model tracks individual humans while mosquito infection dynamics are captured with a compartmental model. The model is age-structured and includes several types of immunity which are gained with exposure and age: immunity against new infections from mosquitoes, immunity controlling parasite densities, detectability, and infectiousness to mosquitoes, and immunity against clinical and severe disease episodes. Malaria control interventions have been included and were parameterized using clinical trials; further details of how interventions were modeled in this study are given below.

The transmission model does not explicitly track parasite densities in the liver or blood and models chemoprevention as having a binary effect on each individual infection event—either preventing the new infection from reaching slide-positive blood-stage infection or not. In order to use the PKPD model output within the transmission model, we converted it into the probability of preventing blood-stage infection on a particular day after treatment as follows (the PKPD model explicitly includes cabamiquine liver-stage action already). Protection against each infectious bite is assumed to be independent if a person receives multiple infectious bites. The time between sporozoite inoculation and observed positive parasitemia in a patient in the PKPD model varies depending on individual variation in PK and PD, and parasite dynamics. From the PKPD simulations, for each day of sporozoite inoculation, we calculated (i) the probability of any future blood-stage infection across patient simulations and (ii) a distribution of duration of time from sporozoite inoculation to blood-stage parasitemia >10 parasites per microliter in those who do have a future blood-stage infection. Summing across all possible days of sporozoite inoculation and times of reaching patent infection in an untreated group versus a treated group, we calculated the protective efficacy on each day against a new blood-stage infection reaching patency, conditional on having been exposed. We counted new superinfections in the protective efficacy as well as first infections.

The resulting protective efficacy over time curves for each dose were input into the transmission model. We also explored different levels of gametocyte-killing activity to represent the potential effect of cabamiquine-pyronaridine on gametocytes. We model gametocyte-killing effects as a reduction in infectiousness in a treated person. As the exact effect on gametocytes is unknown, we explored two assumptions about gametocyte killing. The default is that cabamiquine-pyronaridine has a similar impact on levels of human infectiousness as SP-AQ (68% reduction), and we also explored a 95% reduction in infectiousness in line with other antimalarials that have a greater impact on infectiousness such as artemisinin combination therapies (18, 24).

We simulated different chemoprevention strategies in different transmission settings. We chose three archetypes of transmission setting representative of different areas where chemoprevention may be used: (i) a high transmission setting with highly seasonal transmission representative of the Center region in Burkina Faso, where SMC is currently carried out; (ii) Ashanti region in southern Ghana, a high transmission region with a longer malaria transmission season; and (iii) Nord-Kivu region in DRC, a moderate transmission area with perennial transmission and little seasonal variation. For simplicity and transparency, we assumed no control interventions other than a constant 50% coverage of effective case management using artemether-lumefantrine. All parameters are detailed in Table S2.

### SP-AQ protection

To compare cabamiquine-pyronaridine predicted impacts with the current standard of care, we modeled three scenarios of SP-AQ protection against infection: (1) full adherence and low SP resistance, (2) high SP resistance, and (3) lower adherence and low SP resistance. The protective efficacy of SP-AQ in scenarios 1 and 2 has been previously estimated from fitting to trials of SP-AQ chemoprevention, using a method which adjusts for underlying transmission intensity and estimates the probability of protection against new infections conditional on being exposed (25). Weibull survival curves were found to capture the declining probability of protection over time since treatment, *t*: $e^{-{\left(\frac{t}{\lambda }\right)}^{w}}$, where λ is the scale parameter and w is the shape parameter. For scenario 1, we used an estimate of SP-AQ protection from a trial (26) in an area of low SP resistance (no *pfdhps* K540E or A581G mutations) and moderate AQ resistance in West Africa (pfcrt 76T and pfmdr1 86Y 30–60%), where the average protection over 30 days was 92% (21, 27). This drug profile was previously validated against eight further trials of SMC in West Africa in settings with similar drug resistance levels (28). For scenario 2, we used a second estimate from a trial in Uganda (29) with high SP resistance and low AQ resistance, where the overall protective efficacy was slightly lower at 87.4% (27).

We further extended this work to incorporate SP-AQ adherence for scenario 3. In trials, SP-AQ treatment is often fully supervised by a health worker over the 3 days of treatment, but in routine settings, SP-AQ is often given with only the 1st day of treatment directly supervised by a health worker (SP and one dose of AQ), while the second and third doses of AQ on days 2 and 3 are not supervised. We used data from a trial contrasting fully supervised SP-AQ treatment on all 3 days versus unsupervised second and third doses (30). Here, the prevalence of infection at day 28 was higher in the unsupervised group, at 9.2%, versus 5.8% in the supervised group. We estimated the protection profile of SP-AQ over time in the unsupervised group as follows. A similar but simplified model structure was used as in previous work: in brief, individuals are assumed to clear 100% of existing infections upon SP-AQ treatment and then be subject to a constant incidence of infection Λ in the absence of drug protection, dependent on local transmission intensity. The incidence of infection at time t in individuals who took chemoprevention is therefore:


$$\Lambda e^{-{\left(\frac{t}{\lambda }\right)}^{w}}$$

And the proportion infected, *I,* at time *t* is found by numerically integrating the following ODE:


$$\frac{dI}{dt}=\Lambda e^{-{\left(\frac{t}{\lambda }\right)}^{w}}$$

Firstly, we estimated the incidence of infection in the trial of supervised versus unsupervised individuals. The drug protection parameters were fixed at the values previously estimated in another West African setting with fully observed treatment, and the force of infection was varied to calibrate to the prevalence of infection in the supervised group at day 28 (5.8%), resulting in an estimated value of 1.0 infections per child per month. Next, we estimated what change in drug protection would be required to obtain the observed prevalence of 9.2% in the unsupervised group. Due to a lack of detailed information on time at infection, we fixed the shape parameter to the previously estimated value and varied the scale parameter to fit to the prevalence. Key drug parameters are presented in Table S1.

### Vaccination

The new pre-erythrocytic malaria vaccines now being scaled up in many endemic countries, RTS,S/AS01 and R21/Matrix-M, are targeted at 5- to 17-month-olds. Since this target age group overlaps with the target SMC age group, we additionally introduced vaccination in some scenarios to test how this would modify SMC impact. RTS,S and R21 are included in the ICL transmission model based on model fitting to individual patient data from phase 3 and phase 2 trials, respectively. These vaccine models are described in detail elsewhere (31, 32), but in brief, the model incorporates antibody boosting by the vaccine and a biphasic decline in antibody titers over time since vaccination. The relationship of antibody titers with vaccine efficacy is described using a dose-response relationship. Protection is assumed to begin at the time of the third dose and to be boosted when a fourth dose is received. The model incorporates individual variability in antibody titers.

### Scenarios modeled

The ICL transmission model, described above, incorporates seasonality by allowing mosquito population dynamics to be driven by rainfall patterns (33). In previous work, daily rainfall estimated by CHIRPS was aggregated at the first administrative subnational level for each country and averaged over a number of years. These average patterns were fitted to the CHIRPS rainfall estimates using Fourier transform methods (34). We chose rainfall patterns from three subnational regions representing different levels of seasonality: (i) Center region, Burkina Faso, has strongly seasonal transmission with a long dry season, (ii) Ashanti region, Ghana, has a longer transmission season, and (iii) Nord-Kivu region, DRC, has perennial transmission with little seasonal fluctuation. We varied the annual entomological inoculation rate (EIR) assumed in these settings from ~10 to ~57, only selecting relatively high transmission intensities to capture where SMC is likely to be used. The seasonal patterns in clinical incidence over the year in each setting are shown in Fig. 2.

Chemoprevention was modeled as the distribution of either SP-AQ or cabamiquine-pyronaridine to 100% of the eligible population. The eligible population was modeled as either the standard SMC age: 3 months to 5 years, expansion of SMC to older children covering 3 months to 15 years, or as an MDA to the entire population >3 months old. SP-AQ was distributed once a month for 4 or 5 months, as is the standard recommendation. Cabamiquine-pyronaridine, with a predicted longer duration of action, was given for three to five cycles annually, spaced either 1 month or 6 weeks apart. For each drug regimen and seasonality setting, we calculated the optimal timing for starting SMC to the nearest week based on the highest number of clinical cases averted over the whole year. We also modeled chemoprevention with and without malaria vaccination with RTS,S at 100% coverage with four vaccine doses given at age 5, 6.5, and 8 months plus a booster dose 12 months after the third dose. In the simulation, we introduced vaccination 5 years prior to starting SMC, so that under-5-year-olds would be already vaccinated at the time of chemoprevention.

### Costs

Cost data were taken from an economic analysis of the ACCESS-SMC program in seven countries where SMC was scaled up (Burkina Faso, Chad, Guinea, Mali, Niger, Nigeria, The Gambia; Table S3) (35). The original analysis was done from the program’s perspective and presented costs in 2016 US dollars, broken down by different activities of the SMC implementation. For costing the chemoprevention strategies in this study, we took the median cost across countries for each component and converted costs to 2025 USD using the GDP implicit price deflator from the International Monetary Fund World Economic Outlook (36, 37). We divided the costs into those which are incurred for each SMC cycle (drugs and supplies, per diems, salaries, travel), versus fixed costs which occur regardless of the number of cycles per year (training, meetings, NGO program management, social mobilization, and behavior change communication). We assumed the per-cycle drug costs in the program would remain unchanged in the SP-AQ scenarios modeled. Although the cost of cabamiquine-pyronaridine is unknown, we considered it likely to cost more than SP-AQ, and therefore assumed the drug costs presented in reference 35 would be two or three times higher per cycle in the cabamiquine-pyronaridine scenarios.

R code for transmission modeling used in this analysis is available at https://github.com/lucyokell/caba_pyro_public.

## RESULTS

### Duration of protection provided by cabamiquine

**Fig 1:**
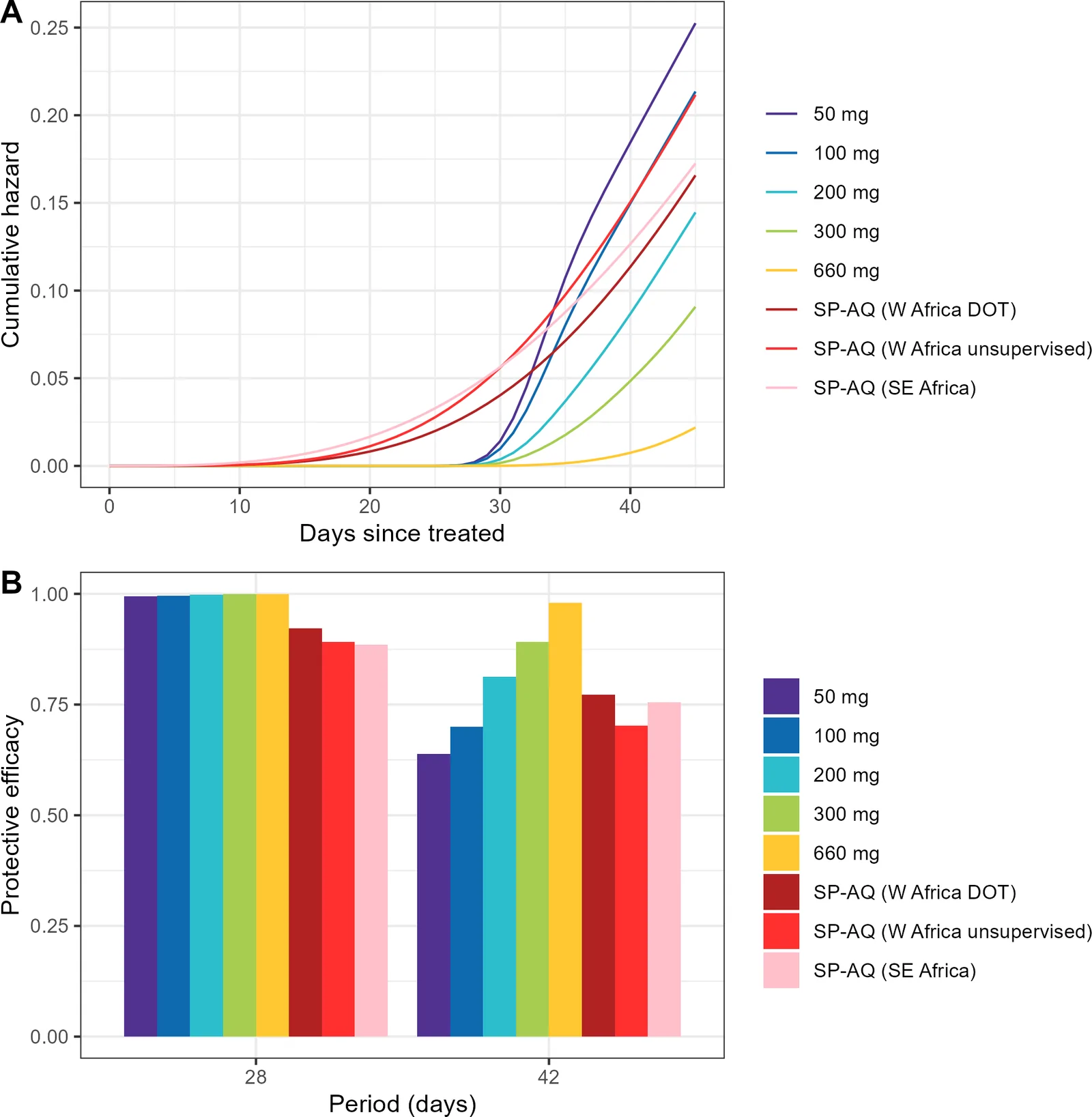
(**A**) Cumulative hazard of new infections per person over time since treatment with different doses of cabamiquine (50 mg–660 mg) and SP-AQ (three scenarios: impact in West Africa with DOT, vs in West Africa with unsupervised treatment, vs in SE Africa). Force of infection in the absence of treatment is set to be, on average, five per person per year. (**B**) Protective efficacy (% of new infections prevented over different time periods). Multiple infections per person are modeled.

The PKPD model analysis predicts that protection by cabamiquine-pyronaridine is high and close to 100% across the different cabamiquine doses over the first 28 days after treatment (Fig. 1). Protection is predicted to increase with higher cabamiquine doses when looking at 42 day outcomes, with predicted protection from 64% with the combination containing 50 mg cabamiquine, up to 98% with 660 mg cabamiquine. Cabamiquine-pyronaridine was estimated to provide similar or slightly better protection than SP-AQ at the 28 day follow-up time across all doses, while at 42 days, only the combinations containing 200 mg cabamiquine and above were predicted to perform better. For example, the 300 mg dose of cabamiquine provides an estimated 89% protection for 6 weeks, versus 70%–77% protection provided by SP-AQ (Fig. 1).

We estimate that the protection provided by SP-AQ when doses 2 and 3 are unsupervised, that is, when SMC participants may not take the full course, is 87% versus 92% of new infections prevented over 30 days with fully observed treatment. The 30 day protective efficacy of SP-AQ in Uganda with higher levels of SP resistance was previously estimated at 87%.

### SMC with cabamiquine-pyronaridine in different epidemiological settings

**Fig 2:**
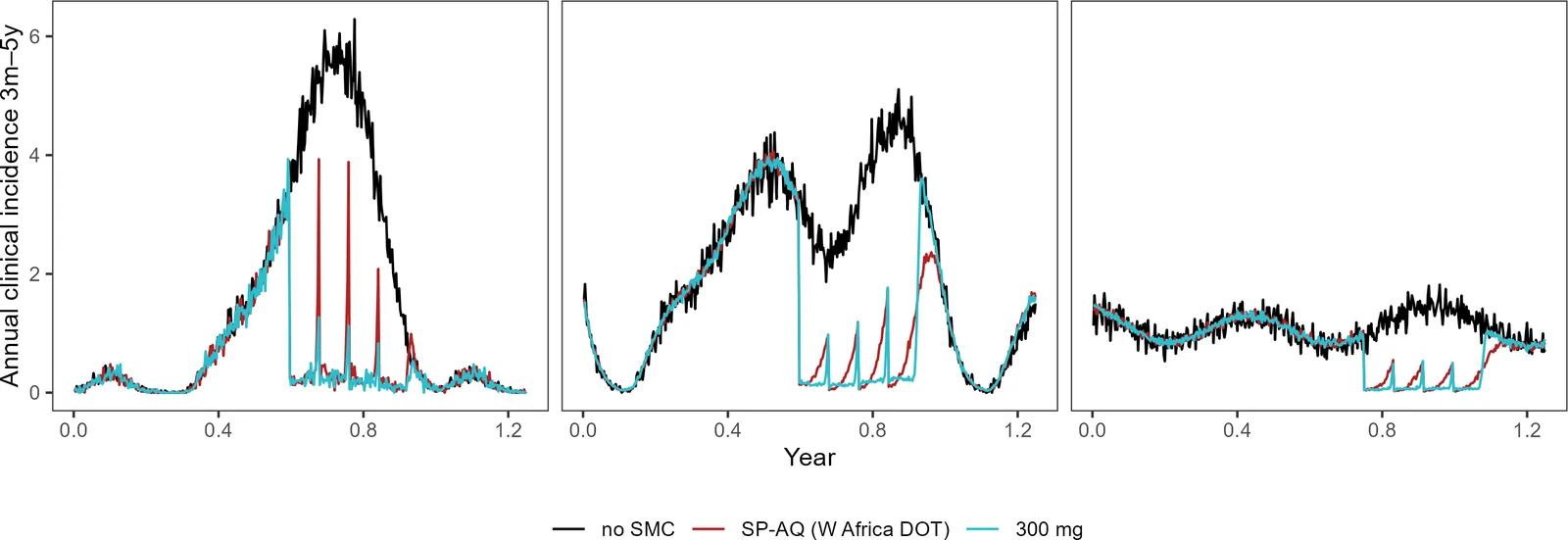
Seasonal pattern in daily clinical incidence in the standard SMC-target age (3 months to 5 years) in the three settings selected for analysis, and the modeled impact of four cycles of SP-AQ (West Africa DOT profile) and cabamiquine-pyronaridine with the 300 mg cabamiquine dose. SMC is given at the times marked by red dotted lines, which are 30 days apart. The impact of other doses is summarized in Fig. 3. See Fig. S1 for slide-prevalence over the season in these settings.

**Fig 3:**
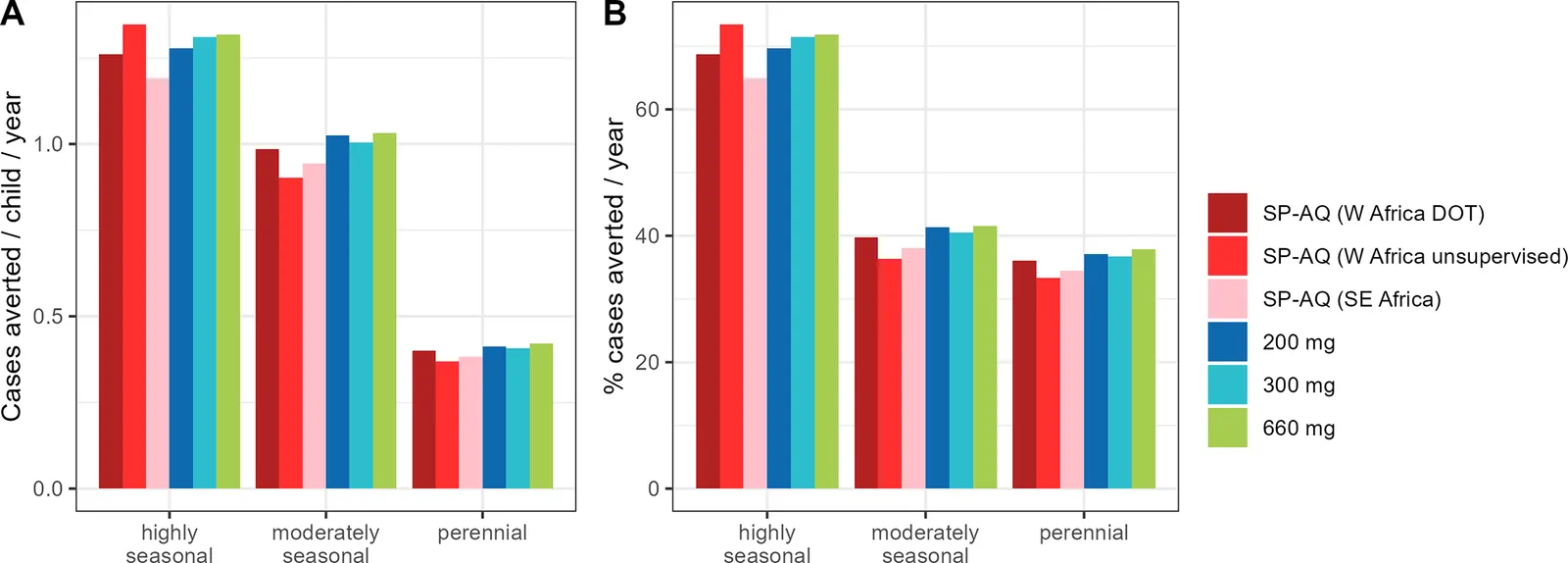
Summary impact on uncomplicated clinical malaria cases over the year from the first dose of SMC, with four cycles 1 month apart: (**A**) cases averted per child per year in the SMC target age group (3 months to 5 years); (**B**) % reduction in cases over 1 year. SP-AQ is contrasted with cabamiquine-pyronaridine (doses of cabamiquine ranging from 200 to 660mg).

In each simulated transmission setting, we show the predicted impact of 4 monthly cycles of SMC with SP-AQ or cabamiquine-pyronaridine (Fig. 2 and Fig. 3). The timing of the start of SMC is optimized for maximum impact for SP-AQ, and we initially model cabamiquine-pyronaridine being given at the same times as this standard SMC program. As expected from the results in the previous section, all doses of cabamiquine-pyronaridine have a similar impact to SP-AQ when given monthly (Fig. 3). Total impact on malaria cases depends on both the transmission intensity and seasonality in the local setting. Chemoprevention is predicted to prevent a greater absolute number of cases in the settings with a higher incidence per child per month during the SMC intervention, simply because of the higher burden. Approximately one to two cases are averted by four SMC cycles with cabamiquine-pyronaridine per child per year in the highly seasonal and moderately seasonal settings, which have a clinical incidence of ~0.25–0.4 cases per child per month during the SMC intervention, versus approximately 0.4–0.5 cases averted per child per year in the lower transmission perennial setting with a clinical incidence of ~0.08–0.16 cases per person-month. However, the percentage reduction in cases is larger in the highly seasonal area, where 4 monthly cycles prevent a large proportion of the annual cases in under-fives (68%; Fig. 3). In the moderately seasonal and perennial transmission (Fig. 2 and 3), four cycles of SP-AQ can only cover a fraction of the transmission season, averting 40% and 36% of cases, respectively.

**Fig 4:**
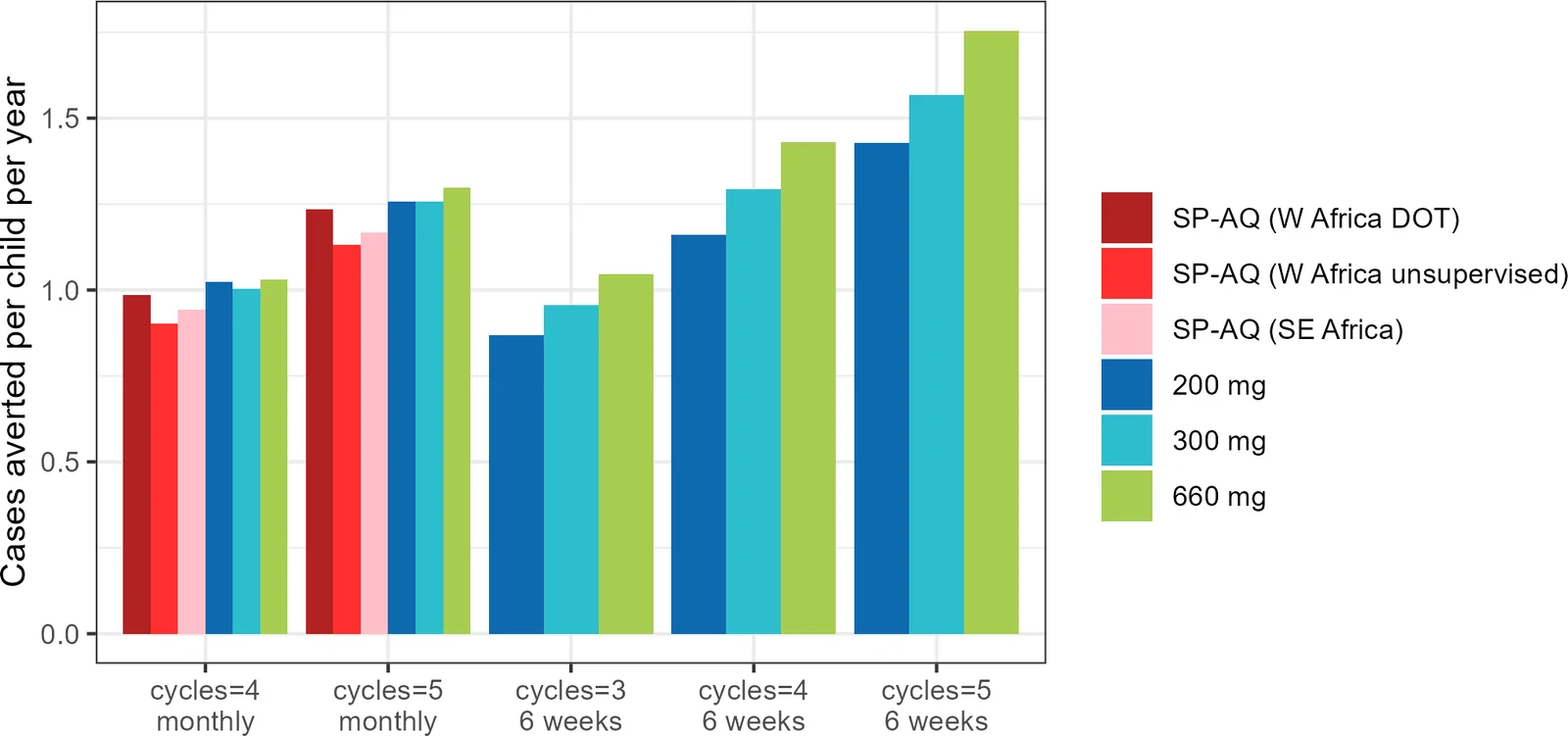
Summary cases averted by SMC in the moderately seasonal setting comparing three, four, or five cycles, and cycles spaced 1 month, 6 weeks, or 2 months apart. We only show monthly spacing for SP-AQ as this is the currently recommended frequency. Cabamiquine-pyronaridine spacing is varied (dosing range 200 mg–660 mg).

Since the 6 week protection provided by cabamiquine-pyronaridine is predicted to be higher than SP-AQ where the cabamiquine dose is ≥200 mg, we explored the potential impact of spacing SMC cycles 6 weeks apart instead of monthly and different numbers of cycles per year (three to five). In the moderately seasonal setting, the higher-dose combinations are predicted to achieve a similar impact to SP-AQ with fewer cycles; for example, three cycles of cabamiquine at doses ≥300 mg are estimated to have a similar impact to the standard 4 monthly cycles of SP-AQ (Fig. 4). The combinations with the highest doses of cabamiquine also have more impact when they are spaced further apart; for example, four cycles of cabamiquine-pyronaridine at doses >200 mg have more impact with 6 week spacing than with monthly spacing. However, this greater impact would need to be offset by assessing risks for resistance with allowing more parasites to be exposed to lower drug levels in between cycles.

### Cost-effectiveness

**Fig 5:**
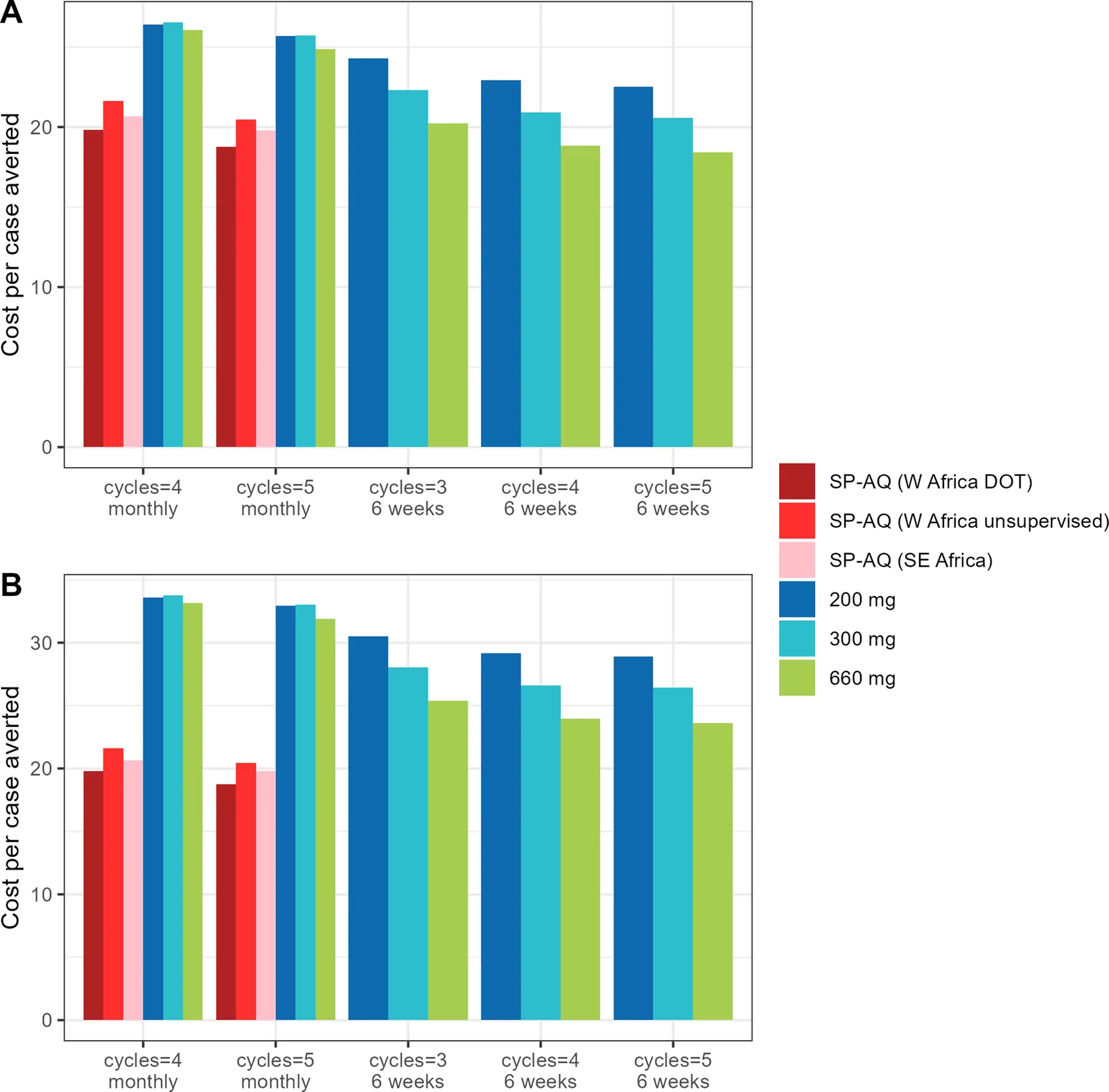
Cost (in 2025 USD) per case averted in the moderately seasonal transmission setting by SMC number of cycles, spacing between cycles, and drug, if the cabamiquine-pyronaridine regimen (cabamiquine dose 200 mg–660 mg) is (**A**) twice or (**B**) three times as expensive as SP-AQ.

In a previous cost analysis across seven countries, a median of 37% of total SMC program costs (range 20%–50%) were due to the cost of drugs. Keeping other costs constant per SMC cycle, we find that if cabamiquine-pyronaridine costs twice as much as SP-AQ, the cost-effectiveness of cabamiquine-pyronaridine would be worse if both were given as monthly cycles. However, if cabamiquine-pyronaridine were given as 6-weekly cycles, the higher doses (200 mg cabamiquine or more) could have a similar cost-effectiveness in terms of cost per case averted in the moderately seasonal transmission setting (Fig. 5). This is because cabamiquine-pyronaridine is predicted to be more long-lasting and could be given as fewer cycles with longer spacing in between. For example, four cycles of the 300 mg dose with 6-weekly spacing have a similar cost-effectiveness to monthly unsupervised SP-AQ (~$21.6 and $20.9 per case averted, respectively). If the cost of the cabamiquine-pyronaridine regimen is three times higher than SP-AQ, it becomes less cost-effective than SP-AQ in most scenarios with a cost-effectiveness of $23–34 per case averted, although the highest dose (660 mg cabamiquine) with wider spacing between cycles is still competitive (Fig. 5B). For comparison, the R21 vaccine costs approximately $34 (16–68) per case averted in USD 2023 in a moderate transmission setting (31).

### Relative impact of SMC is maintained in the presence of vaccine

**Fig 6:**
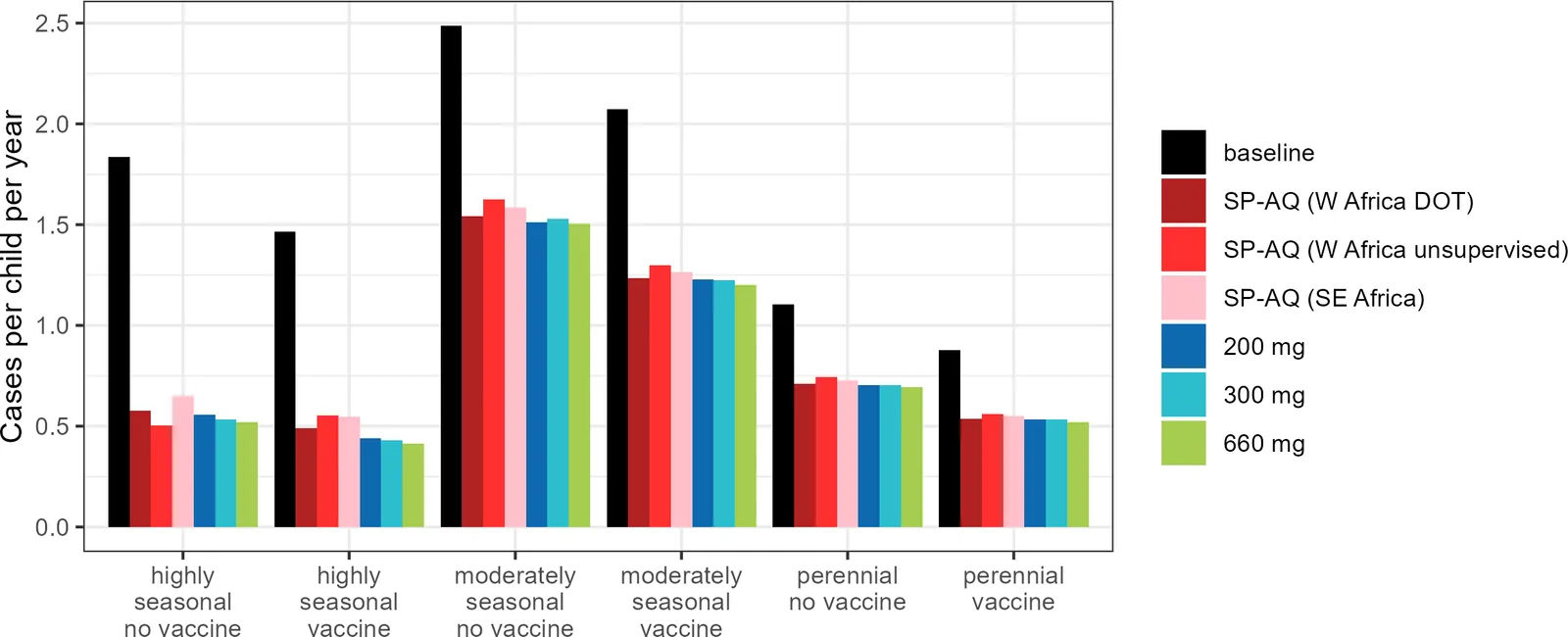
SMC impact in the presence versus absence of vaccine. Incidence of uncomplicated malaria cases per child in the SMC target age group (3 months to 5 years) in the presence and absence of vaccine and with and without different SMC regimens (SP-AQ and cabamiquine-pyronaridine 200 mg–660 mg dose range). All SMC regimens were implemented as four cycles per year with monthly spacing and 100% coverage. Vaccine was implemented at 100% coverage.

The RTS,S vaccine is predicted to lower the annual cases in the 0- to 5-year-old age group by 17%–21% in the example settings in simulations where all under-5-year-olds have been previously vaccinated, if implemented at 100% coverage (Fig. 6). This prediction is based on former model calibration to the phase 3 RTS,S trial (38). We estimate that SMC causes a near-identical percentage reduction in cases in settings with and without vaccination. Therefore, the vaccine simply reduces the baseline transmission, and the absolute number of cases averted is also reduced by 17%–21%. For example, in the moderately seasonal transmission setting simulated here, SMC with four cycles of cabamiquine-pyronaridine (300 mg pyronaridine) is estimated to reduce annual cases by 40.5% in the absence of vaccine and by 42.3% in the presence of vaccine. However, the starting incidence in the absence of vaccine is 2.5 episodes per child per year, versus 2.1 in the presence of vaccine. Therefore, SMC is estimated to avert 1.00 episodes per child per year in the absence of vaccine, and 0.88 in the presence of vaccine.

### Impact on population-level transmission

Chemoprevention can have indirect effects on total population transmission if given to a large enough proportion of the population, that is, by reducing infection in the target group, chemoprevention can reduce prevalence and incidence in the population outside the target age group. In some trials or implementation programs, certain countries have expanded SMC to older age groups, for example, 3 months to 10 years in Senegal (39), deployed chemoprevention in school-age children (40), or given MDA to the whole population of any age (usually excluding very young infants and pregnant women) (41). Furthermore, cabamiquine shows potential gametocyte-killing activity (42), which might enhance these population-level effects.

We estimated the impact of chemoprevention strategies on transmission (Fig. S2). SMC with cabamiquine-pyronaridine given to children under 5 years has a small and short-lived effect on the EIR (the rate of infectious bites per person). This is because the age group represents a relatively small proportion of the infectious reservoir in the model (which was calibrated against age-infectivity data), and after the drug effect wanes, transmission rapidly returns to its previous equilibrium conditions. SMC targeted at children up to 15 years of age has a much larger effect on population transmission, with the peak EIR approximately halved for a number of months before returning to normal. The full population MDA has the largest effect, which is predicted to reduce EIR into the next transmission season.

Assuming high levels of gametocyte-killing activity by cabamiquine has little predicted additional impact on population-level EIR when the drug is used for SMC or extended-age SMC, but has a more noticeable impact for an MDA, where transmission in the following year returns more slowly (Fig. S3).

## DISCUSSION

Our analysis has used mathematical models to translate the early results of phase I clinical trials of cabamiquine, when combined with pyronaridine, into estimates of its potential impact as a chemoprevention drug against *P. falciparum* malaria. Uncertainties of this analysis must be acknowledged. Firstly, the phase I trials had a relatively small number of participants for characterizing the PK and PD profiles of cabamiquine and pyronaridine. The trials were done in healthy, malaria-naïve adults, and the PKPD relationship may be different in malaria patients in endemic settings, who are largely children. Also, in these trials, cabamiquine and pyronaridine were given either after or very shortly before the experimental inoculation of parasites. To predict the impact of chemoprevention, we estimated what would happen if a single oral dose of cabamiquine-pyronaridine had been given a longer time prior to inoculation of parasites, which could occur at any time after treatment. A strength of the mathematical modeling methods is this ability to extrapolate integrated PKPD effects to predict impact in multiple possible scenarios (i.e., many different infection time points), but a limitation is that we could not yet validate this prediction against clinical data. We assumed independent drug action of cabamiquine and pyronaridine, apart from potentiation of pyronaridine on the cabamiquine liver-stage action (16), but drug interactions will need to be fully confirmed. Phase 2 trials for cabamiquine-pyronaridine are recently completed, and we aim to validate the accuracy of the model predictions against new data as it becomes available.

While there are caveats to our analysis, the early predictions for a single oral dose of cabamiquine-pyronaridine chemoprevention appear promising. At the higher doses of 200 mg or more, cabamiquine-pyronaridine is predicted to provide longer chemoprevention than SP-AQ, with, for example, the 660 mg dose providing a protective efficacy of 97.8% over 8 weeks, versus 62% for SP-AQ. It may therefore be possible to reduce the frequency of chemoprevention or the total number of cycles given per year while achieving the same impact. This increased efficiency may offset some increase in the price of the chemoprevention drug, that is, given that most new drugs are likely to be more expensive than SP-AQ, especially as around 63% of the average SMC program cost is non-drug expenses. Compared with SP alone, which is currently used in some PMC programs, the advantage of cabamiquine would be greater, with SP protective efficacy at 1 month being as low as 15% in areas with the *pfdhps* 437-540-581 mutant (43). The spread of mutations in the parasite *dhfr* and *dhps* genes also means that the future lifespan of SP-AQ is uncertain, particularly in southern and eastern Africa. Resistance to AQ has become lower in the region but could become a threat to SP-AQ efficacy if it returns. SP-AQ may need replacing in the coming years if resistance worsens.

While the dose ratio combination is still being finalized, cabamiquine-pyronaridine has successfully completed phase 2a trials as a single dose (12) and could therefore have a further advantage over SP-AQ, which requires adherence to three doses to achieve the full effect. Here, we used data from one trial (30) comparing supervised to unsupervised SP-AQ to estimate the loss of effectiveness due to incomplete adherence. We found relatively little drop in impact with unsupervised treatment. However, initial results of a more recent trial (44) appeared to find a larger drop in effectiveness in unsupervised compared to supervised treatment. If this is confirmed, it will be important for future analyses to factor in either a lower impact of unsupervised SP-AQ or the costs of directly observed treatment. Furthermore, the future cost of cabamiquine-pyronaridine remains unknown. As with other chemopreventive interventions, the cost-effectiveness of this strategy will need to be assessed relative to other malaria control measures in children, particularly vaccination, which has recently become more affordable following cost reductions (45). Our predictions of a multiplicative effect of SMC and vaccine combined are broadly in line with trials that assessed SMC and vaccine alone versus together (21). We do not, however, capture the potential different reach of the two interventions into different groups of children, since we simulate both interventions at 100% coverage. In reality, some children who do not receive the vaccine might be more likely to receive SMC, and vice versa.

In future, it will be important to consider the risk of evolving drug resistance in the context of using cabamiquine-pyronaridine for chemoprevention. Early *in vitro* studies have identified cabamiquine resistance-associated mutations in laboratory strains and clinical isolates (46); however, these strains are pyronaridine-sensitive. Dose predictions have been carefully modeled based on both susceptible and resistant strains (47). Monitoring such mutations during future trials will further inform optimal cycle frequency and dosing to reduce the risk of resistance. No resistance against pyronaridine has yet been described (48). We found that cabamiquine has some potential to impact population transmission if given to expanded age groups; although, as with any antimalarial drug, the effect is temporary and transmission is predicted to return to previous levels relatively rapidly in high transmission areas (49). Treatment would need to be given repeatedly and combined with other interventions to have a sustained impact on transmission.

In this work, we show that integrated modeling can provide early, quantitative estimates of the potential impact of a drug candidate and thereby inform development decisions well before large clinical phase III trials. Such models also have potential to inform trial design, for example, informing sample sizes and power calculations. A critical next step, however, is prospective validation of the model against clinical trial data generated in malaria-endemic settings, which will be essential to assess and confirm the accuracy and generalizability of the predictions.

## SUPPLEMENTAL MATERIAL

Supplemental material aac.00434-26-s0001.docx Fig. S1 to S4; Tables S1 to S3.

ASM does not own the copyrights to Supplemental Material that may be linked to, or accessed through, an article. The authors have granted ASM a non-exclusive, world-wide license to publish the Supplemental Material files. Please contact the corresponding author directly for reuse.
