# Supplementary material for: Modeling the potential effect of cabamiquine-pyronaridine for chemoprevention against Plasmodium falciparum malaria
Source: Antimicrob Agents Chemother. 2026 Oct 8;70(11):e00434-26. doi: 10.1128/aac.00434-26 (PMC13731186; doi:10.1128/aac.00434-26)
Supplement: Supplementary file 1 [file aac.00434-26-s0001.docx]

Supplementary Information: Modelling the potential effect of cabamiquine-pyronaridine for chemoprevention against *Plasmodium falciparum* malaria

Lucy C Okell^1^*, Perrine Courlet^2^, Filippo Venezia^3^, Claude Oeuvray^4^, Claudia Demarta^4^, Thomas Spangenberg^4^, Joseph Okebe^4^

Affiliations

^1^MRC Centre for Global Infectious Disease Analysis, School of Public Health, Imperial College London

^2^Merck Institute for Pharmacometrics, Ares Trading S.A., (an affiliate of Merck KGaA, Darmstadt, Germany), Lausanne, Switzerland

^3^The healthcare business of Merck KGaA, Darmstadt, Germany.

^4^Global Health R&D of Merck Healthcare, Ares Trading S.A. (an affiliate of Merck KGaA, Darmstadt, Germany), Eysins, Switzerland

**Supplementary figures**

**Supplementary Figure S1**. Seasonal patterns in slide prevalence in the standard SMC-target age (3 months – 5 years) in the three settings selected for analysis.

**Supplementary Figure S2** Impact of 4 cycles of monthly chemoprevention on population-wide annual EIR over time, when given to different age groups in the moderately seasonal transmission setting.

**Supplementary Figure S3** Gametocyte-killing effects. Impact of 4 cycles of monthly cabamiquine-pyronaridine with lower or higher gametocyte-killing effects on population-wide annual EIR over time, when given to different age groups in the moderately seasonal transmission setting. We show the 660 mg dose of cabamiquine, and ‘gam’ denotes higher gametocyte killing activity. We assume the dosage only affects the duration of prophylaxis, not the gametocytocidal effects.

**Supplementary Figure S4** Integrated population pharmacokinetic-parasitemia model linking cabamiquine pharmacokinetics ^13^, liver-stage parasite dynamics, blood-stage parasite dynamics, and pyronaridine pharmacokinetics ^14^. Cabamiquine was assumed to drive parasite killing in both liver and blood stages, while pyronaridine contributed directly to blood-stage killing. Combined blood-stage activity of cabamiquine and pyronaridine was modeled as a two-sided additive Bliss independence framework with negligible-overlap approximation, whereas the liver-stage effect was captured phenomenologically as enhanced apparent cabamiquine potency in the presence of pyronaridine.

***C_L_****, systemic clearance;* ***C_ab_****, cabamiquine concentration in the central compartment;* ***C_p_****, pyronaridine concentration in the plasma compartment;* ***C_pt_****, pyronaridine concentration in the tissue compartment;* ***k_a_****, absorption rate constant;* ***k_g1_****, depot-to-absorption transfer rate constant;* ***k_2g_****, central-to-depot transfer rate constant;* ***k_tr_****, transit rate constant;* ***MTT****, mean transit time;* ***M_TIME_****, depot emptying time.* ***V_2_****, central volume of distribution for cabamiquine;* ***V_3_****, peripheral volume of distribution 1;* ***V_4_****, peripheral volume of distribution 2;* ***V_p1_****, central (plasma) volume of distribution for pyronaridine;* ***V_p2_****, peripheral (tissue) volume of distribution for pyronaridine;* ***Q_2_****, intercompartmental clearance between central and peripheral compartment 1;* ***Q_3_****, intercompartmental clearance between central and peripheral compartment 2;* ***Q_p_****, intercompartmental clearance between pyronaridine plasma and tissue compartments.* ***F_inc_****, fraction of infectious inoculated sporozoites invading hepatocytes;* ***inoculum****, number of injected sporozoites;* ***k_gr,l_****, parasite growth rate in the liver stage;* ***k_gr,b_****, parasite growth rate in the blood stage;* ***klb****, transition rate from liver to blood stage;* ***T_rlb_****, cumulative fraction of hepatocytes releasing merozoites;* ***T_50_****, time at which 50% of infected hepatocytes have released merozoites;* ***σ_lb_****, dispersion parameter governing the release profile.* ***k_kill,l_****, parasite killing rate in the liver stage;* ***k_kill,b_****, parasite killing rate in the blood stage;* ***K_kill,p_****, pyronaridine-mediated parasite killing rate in the blood stage.* ***E_MAXA_*** *and* ***E_MAXB_****, maximal effects of cabamiquine and pyronaridine, respectively;* ***η_A_*** *and* ***η_B_****, normalized drug-effect functions for cabamiquine and pyronaridine, respectively;* ***ψ_min,AB_****, interaction term in the two-sided Bliss independence model.*

**Supplementary Tables**

**Supplementary Table S1** Key model drug parameters used across all sites

| Parameter description | Name within malariasimulation R package^22^ | Value | Reference |
| --- | --- | --- | --- |
| ACT coverage among uncomplicated malaria cases (%) | clinical_treatment_coverage | 50 | - |
| SP-AQ protection in low resistance sites in West Africa |  |  |  |
| Scale parameter | drug_prophylaxis_scale | 39.34 | ^21^ |
| Shape parameter | drug_prophylaxis_shape | 3.4 |  |
| SP-AQ protection in high SP resistance site in Uganda |  |  |  |
| Scale parameter | drug_prophylaxis_scale | 40.8 | ^27^ |
| Shape parameter | drug_prophylaxis_shape | 2.28 |  |
| SP-AQ protection when unsupervised (lower adherence) |  |  |  |
| Scale parameter | drug_prophylaxis_scale | 33.6 | This paper |
| Shape parameter | drug_prophylaxis_shape | 3.4 |  |

**Supplementary Table S2** Site-specific parameters used in the ICL model malariasimulation package^22^ for baseline transmission before introducing chemoprevention.^22^

|  | Highly seasonal | Moderately seasonal | Perennial |
| --- | --- | --- | --- |
| Initial EIR before treatment introduction (infectious bites per person per year) | 28.4 | 57.3 | 10.7 |
| Seasonality parameters (Fourier coefficients) |  |  |  |
| g0 | 2.46 | 3.92 | 4.74 |
| g1 | -3.2 | -2.13 | 0.33 |
| g2 | 0.44 | -1.9 | -0.74 |
| g3 | 0.73 | -0.55 | 0.13 |
| h1 | -1.82 | -0.99 | -0.48 |
| h2 | 1.4 | -0.86 | -1.14 |
| h3 | -0.33 | 1.36 | -0.03 |

**Supplementary Table S3** Cost data from ACCESS SMC programme, in 2016 US dollars. Costs are categorized as to whether they would be incurred per cycle or as a fixed annual cost regardless of chemoprevention cycle numbers.

|  | **Burkina Faso** | **Chad** | **Guinea** | **Mali** | **Niger** | **Nigeria** | **The Gambia** | Per cycle cost? |
| --- | --- | --- | --- | --- | --- | --- | --- | --- |
| SMC drugs and supplies | 1.24 | 1.16 | 1.2 | 1.23 | 1.26 | 1.2 | 1.57 | yes |
| Distributor per diem and travel | 0.78 | 0.65 | 0.49 | 0.39 | 0.41 | 0.51 | 1.45 | yes |
| Direct supervisor per diem and travel | 0.19 | 0.24 | 0.03 | 0.13 | 0.08 | 0.24 | 0.16 | yes |
| Other supervisor per diem and travel | 0.11 | 0.11 | 0.25 | 0.2 | 0.1 | 0.11 | 0.92 | yes |
| Distributor salaries | 0.07 | 0 | 0 | 0.35 | 0 | 0.15 | 0 | yes |
| Supervisor salaries | 0.1 | 0.15 | 0.09 | 0.1 | 0.09 | 0.31 | 0.06 | yes |
| Meetings | 0.11 | 0.29 | 0.08 | 0.11 | 0.01 | 0.08 | 0.05 | no |
| Training | 0.19 | 0.05 | 0.34 | 0.12 | 0.1 | 0.27 | 0.67 | no |
| NGO programme management | 0.15 | 0.06 | 0.32 | 0.26 | 0.14 | 0.25 | 1.56 | no |
| Social mobilisation and behaviour change communication | 0.1 | 0.18 | 0.26 | 0.2 | 0.07 | 0.09 | 1.31 | no |
| Other costs | 0.05 | 0.05 | 0.2 | 0.04 | 0.09 | 0.09 | 0.12 | no |
| Programme management salaries | 0.29 | 0.17 | 0.12 | 0.03 | 0.16 | 0.25 | 0.11 | no |
